# The natural history and ecology of melanism in red wolf and coyote populations of the southeastern United States – evidence for Gloger’s rule

**DOI:** 10.1186/s40850-022-00138-5

**Published:** 2022-06-20

**Authors:** Joseph W. Hinton, Kyla M. West, Daniel J. Sullivan, Jacqueline L. Frair, Michael J. Chamberlain

**Affiliations:** 1 Wolf Conservation Center, 7 Buck Run, South Salem, NY 10590 USA; 2 Washington Department of Fish and Wildlife, 1111 Washington Street SE, Olympia, WA 98501 USA; 3 United States Environmental Protection Agency, Great Lakes Toxicology and Ecology Division, 26 Martin Luther King Drive West, Cincinnati, OH 45268 USA; 4 Department of Environmental and Forest Biology, State University of New York College of Environmental Science and Forestry, 1 Forestry Drive, Syracuse, NY 13210 USA; 5 Warnell School of Forestry and Natural Resources, University of Georgia, 180 E Green Street, Athens, GA 30621 USA

**Keywords:** *Canis latrans*, *Canis rufus*, Coyote, Gloger’s rule, Habitat selection, Melanism, Red wolf

## Abstract

**Background:**

Gloger’s rule postulates that animals should be darker colored in warm and humid regions where dense vegetation and dark environments are common. Although rare in *Canis* populations, melanism in wolves is more common in North America than other regions globally and is believed to follow Gloger’s rule. In the temperate forests of the southeastern United States, historical records of red wolf (*Canis rufus*) and coyote (*Canis latrans*) populations document a consistent presence of melanism. Today, the melanistic phenotype is extinct in red wolves while occurring in coyotes and red wolf-coyote hybrids who occupy the red wolf's historical range. To assess if Gloger’s rule could explain the occurrence and maintenance of melanistic phenotypes in *Canis* taxa, we investigated differences in morphology, habitat selection, and survival associated with pelage color using body measurements, GPS tracking data, and long-term capture-mark-recapture and radio-telemetry data collected on coyotes and hybrids across the southeastern United States.

**Results:**

We found no correlation between morphometrics and pelage color for *Canis* taxa. However, we observed that melanistic coyotes and hybrids experienced greater annual survival than did their gray conspecifics. Furthermore, we observed that melanistic coyotes maintained larger home ranges and exhibited greater selection for areas with dense canopy cover and wetlands than did gray coyotes.

**Conclusions:**

In the southeastern United States, pelage color influenced habitat selection by coyotes and annual survival of coyotes and hybrids providing evidence that Gloger’s rule is applicable to canids inhabiting regions with dense canopy cover and wetlands. Greater annual survival rates observed in melanistic *Canis* may be attributed to better concealment in areas with dense canopy cover such as coastal bottomland forests. We suggest that the larger home range sizes of melanistic coyotes may reflect the trade-off of reduced foraging efficiency in lower quality wetland habitat for improved survival. Larger home ranges and differential use of land cover by melanistic coyotes may facilitate weak assortative mating in eastern coyote populations, in which melanistic animals may have lower success of finding compatible mates in comparison to gray conspecifics. We offer that our observations provide a partial explanation for why melanism is relatively low (< 10%) but consistent within coyote populations throughout southeastern parts of their range.

## Background

In mammals, pelage color has been linked to fitness-relevant traits such as crypsis, sexual behavior, fecundity, aggressiveness, and immunity [1–5]. Gloger’s rule, the primary ecogeographical rule on animal coloration, postulates that animals should be darker colored in warm and humid regions because these climatic conditions promote dense vegetation and darker environments [6, 7]. To improve camouflage, animals inhabiting humid environments with dense canopy may acquire darker colors than conspecifics inhabiting drier, non-forest habitats. For example, melanism (black pelage color) is observed in felids living in tropical forests, such as melanistic jaguars (*Panthera onca*) and leopards (*Panthera pardus*). However, melanism is rare in other carnivores such as *Canis*, in which black pelage color occurs most commonly in North American gray wolves (*Canis lupus*) [8, 9] and is believed to follow Gloger’s rule [10]. Melanistic gray wolves are rare in Eurasia, but some isolated occurrences of melanism have been documented [11–13]. Collectively, most research aimed at elucidating melanistic traits in *Canis* has focused on the origin and function of melanism in North American gray wolves [9, 14–16] and to a lesser extent, Eurasian wolves [17, 18].

**Fig. 1:**
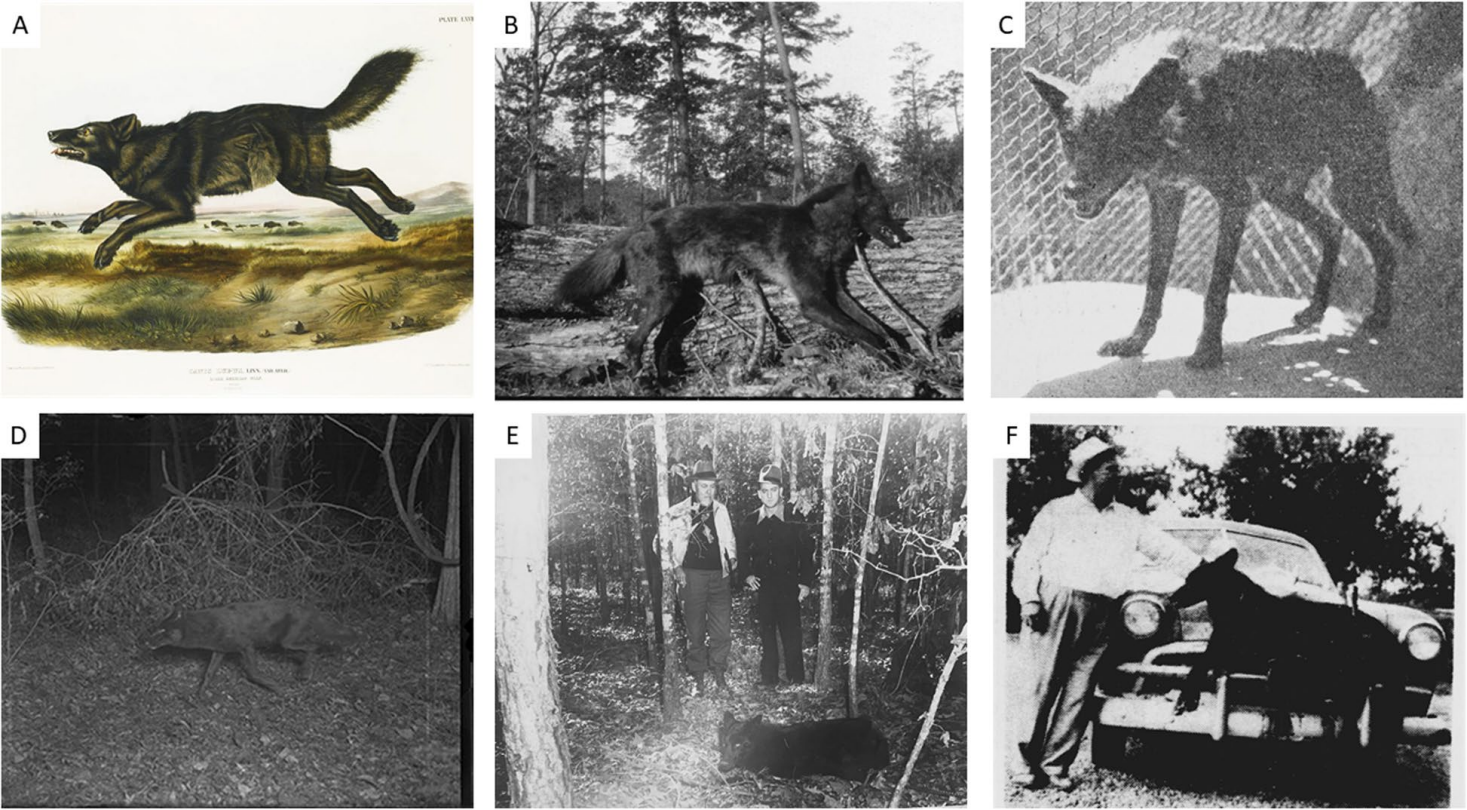
**A** “Black American Wolf”, a nineteenth-century hand-colored lithograph drawn from a Florida specimen [20]. **B** A melanistic red wolf killed in Oklahoma, May–June 1919. Reverend James O. Arthur photograph collection from the National Museum of the American Indian repository at the Smithsonian Institute. **C** Melanistic red wolf, taken from Evangeline Parish and exhibited in the Audubon Park Zoo in New Orleans, LA in the late 1920s. Photo featured in *The Fur Animals of Louisiana*, 1931 by Stanley Clisby Arthur. **D** A melanistic red wolf in Tensas Parish, Louisiana, 1934. Copyright Tappan Gregory (1886–1961). **E** Melanistic wolf that weighed 32.2 kg. Shown in the trap in Winn Parish, Louisiana, 1948. Courtesy of the T.E. “Doc” Harris family. **F** Photo of a large melanistic wolf killed in Caldwell Parish, Louisiana, 1949 by R.E. Walters. Photo appeared in *The Richland Beacon News*, August 20, 1949

**Fig. 2:**
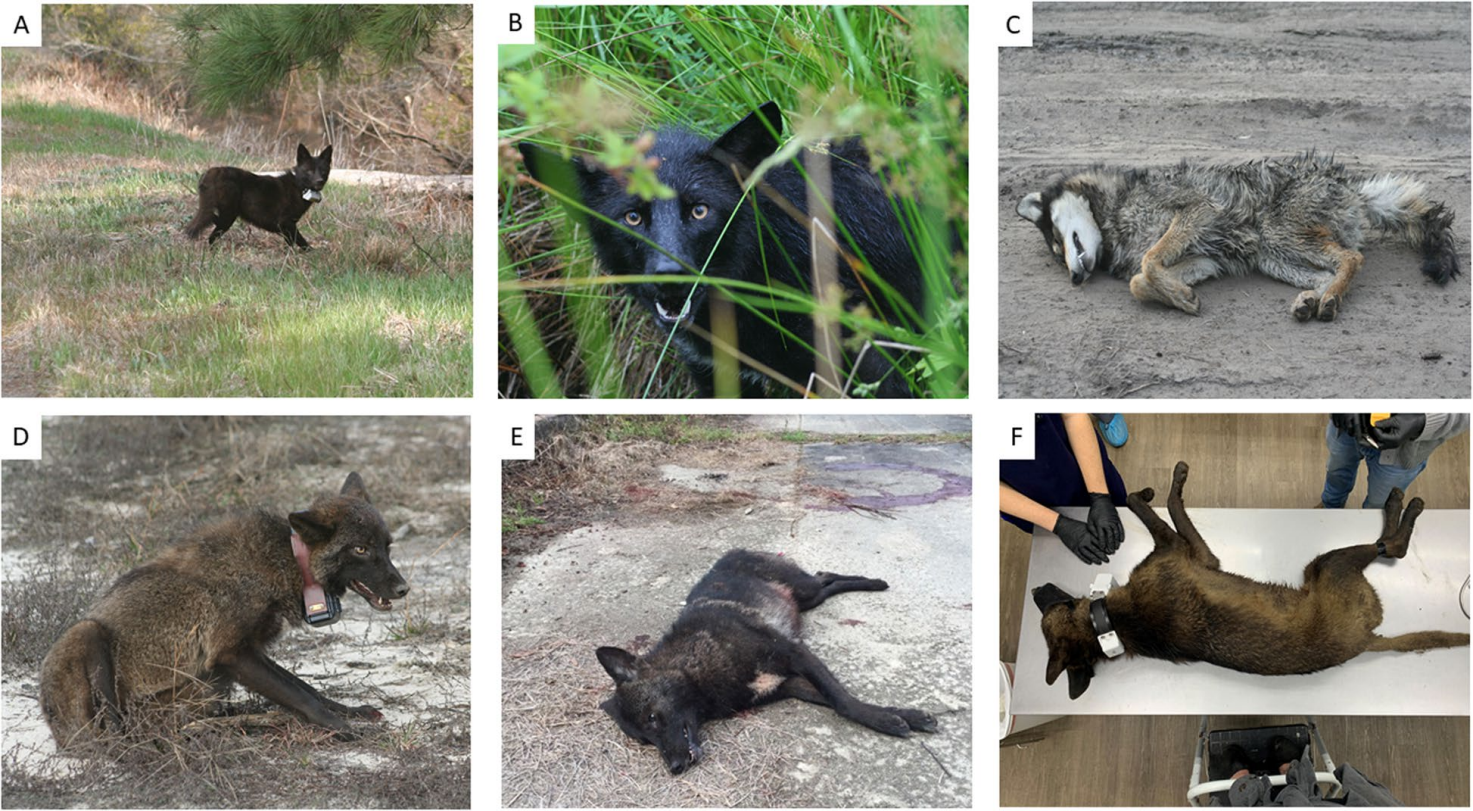
**A** A melanistic coyote (458 M) captured and radio-collared in Tyrrell County, NC, 2009. Photo by Joseph W. Hinton. **B** A melanistic coyote (586 M) captured and radio-collared in Tyrrell County, NC, 2010. Photo by Joseph W. Hinton. **C** A melanistic coyote (634 M) captured in Washington County, NC, 2011 and euthanized at the behest of the landowner. Photo by Joseph W. Hinton. **D** A melanistic coyote (SC34M) captured and radio-collared in Saluda County, SC, 2016. Photo by Joseph W. Hinton. **E** Male melanistic coyote shot by a landowner in Livingston Parish, Louisiana, 2018. Photo courtesy Amy C. Shutt. **F** A melanistic coyote (LA51M) captured and radio-collared in Cameron Parish, Louisiana, 2022. Photo by Amy C. Shutt

Historical and current records demonstrate a consistent presence of melanism in *Canis* populations of temperate forests of the southeastern United States (hereafter Southeast) [19–25] (Figs. 1 and 2). Historically, melanism was common in the red wolf (*Canis rufus*), the only wild *Canis* species that occurred in the Southeast from the terminal Pleistocene until the early twentieth century [23, 26]. Indeed, melanistic wolves were so distinguishable in the Southeast that Goldman [21] combined all red wolf forms into a single species, *Canis niger*. However, the red wolf suffered a severe population bottleneck because of government-sponsored eradication campaigns that resulted in the eventual extirpation of the species from the wild and the use of 14 individuals to establish a captive-breeding program to prevent extinction [27, 28]. Currently, the melanistic phenotype is extinct within the extant red wolf population because only russet-colored individuals were included as founders for the captive population (USFWS, unpublished data). However, melanism occurs in contemporary coyote (*Canis latrans*) populations that replaced red wolves throughout the Southeast [22, 24, 25].

Following their colonization of eastern North America, coyotes experienced increased geographic variation in phenotypic and genetic traits [29]. Melanistic pelage, noticeably absent from western coyote populations, is unique to eastern coyote populations and more commonly found in populations of the Southeast [23–25]. The origin of melanism in eastern coyotes is linked with hybridization with other *Canis* taxa including dogs (*Canis lupus familiaris*) [9], eastern wolves (*Canis lycaon*) [30], and red wolves [22, 23]. Despite the coyote's widespread co-occurrence with gray wolves and dogs in central and western North America, only one case of melanism was reported in Colorado during the 1920s via Young and Jackson’s [31] extensive survey of coyote populations prior to the coyote’s colonization of the Southeast. However, reports of melanistic coyotes became more common in areas of the Southeast where they colonized areas inhabited by melanistic red wolves during 1940–1975 [22, 23, 31–33]. Several studies conducted between 1976 and 2015 indicated that melanistic coyotes were uncommon in the Southeast and comprised 2–9% of the surveyed populations [22, 24, 25]. Recently, Caudill and Caudill [25] concluded that Gloger’s rule did not hold true for coyotes in Florida because they could not conclude that melanistic traits were under selective pressure in the temperate forests of the state.

Given their broad geographic range across North America, coyotes are an ideal species to explore and test ecogeographical rules believed to influence geographic variation in phenotypic and genotypic traits of animal populations [29]. For example, geographic distribution of body mass in coyote populations does not follow Bergmann’s rule because of coyote hybridization with wolves in eastern North America facilitated longitudinal variation in mass rather than latitudinal variation as predicted by Bergmann’s rule [29, 34]. However, longitudinal variation of melanism in coyote populations does not violate Gloger’s rule, because, as observed in other large carnivores such as leopards that exhibit similar west-to-east gradients in melanism [35], factors influencing melanism are associated with canopy cover and humidity rather than colder climates associated with increasing latitude [7].

It is commonly argued that, following hybridization events during colonization of eastern North America, the eastern coyote’s phenotype reflected an adaptive response to larger prey, specifically white-tailed deer (*Odocoileus virginianus*) [34, 36–39]. However, given the near extirpation of white-tailed deer from eastern North America during the early stages of coyote colonization and the coyote’s regular use of ungulates in western North America, Hinton et al. [29] suggested that increased body size improved coyote dispersal capabilities necessary for improving connectivity among coyote metapopulations on the colonization front. If hybridization influenced coyote movement behaviors, we should expect melanism, a conspicuous trait associated with hybridization, to be associated with space use behaviors such as larger home range sizes and increased selection for canopy and wetland cover. Therefore, our objectives were to describe the occurrence, morphometrics, and spatial ecology of melanistic individuals in extant wild populations of *Canis* in the Southeast. We believe examining melanism at a regional scale may provide interesting insight into differences in morphology and behavior associated with pelage color of *Canis* taxa and help explore hypotheses that best explain occurrence and maintenance of melanistic traits such as Gloger’s rule. We used morphometrics to test the hypothesis that melanistic coyotes and hybrids were larger than their gray conspecifics. We used global positioning system (GPS) radiotelemetry data to quantify habitat selection by southeastern coyotes to test the hypothesis that melanistic individuals would exhibit stronger selection for areas with greater canopy and wetland cover than would gray conspecifics. To test the hypothesis that melanistic individuals experience greater survival than gray individuals, we used long-term monitoring data on coyotes and red wolf-coyote hybrids collected by the United States Fish and Wildlife Service (USFWS) Red Wolf Recovery Program (hereafter Recovery Program). Finally, we discuss which mechanisms most likely influence patterns of variation in *Canis* coat color under the context of Gloger’s rule.

## Results

### Morphometrics

Overall, 460 coyotes, 532 red wolves, and 160 hybrids were captured and measured between 1987 and 2016. Morphometric measurements differed among coyotes, red wolves, and hybrids with hybrid measurements falling between the larger red wolf and smaller coyote (Tables 1 and 2). As expected, no red wolves were melanistic, whereas 5.7% of coyotes and 8.5% of hybrids consisted of melanistic individuals.

**Table 1:** Means (± SD) and ranges for body mass and measurements of red wolves, coyotes, and hybrids in the southeastern United States

*Canis* type	Mass (kg)			Body length (cm)			Shoulder height (cm)			Hind foot length (cm)		
	*n*	Mean	Range	*n*	Mean	Range	*n*	Mean	Range	*n*	Mean	Range
Red wolf	484	23.7 ± 4.6	10.1–38.6	444	106.7 ± 6.6	83.5–125.0	442	67.2 ± 3.6	53.5–77.2	448	22.4 ± 1.2	17.0–27.0
Male	252	25.5 ± 4.6	10.5–38.6	233	108.8 ± 6.6	90.5–125.0	233	68.9 ± 3.2	57.7–77.2	236	22.9 ± 1.1	19.6–27.0
Female	232	21.8 ± 4.0	10.1–34.7	211	104.5 ± 6.2	83.5–120.5	209	65.3 ± 2.9	53.5–73.5	212	21.8 ± 1.0	17.0–24.5
Gray coyote	356	13.9 ± 2.0	6.9–20.0	364	90.5 ± 5.4	64.0–106.7	365	56.3 ± 3.5	44.0–67.0	371	18.9 ± 1.1	16.0–22.5
Male	182	14.6 ± 0.2	6.9–20.0	180	91.9 ± 5.8	64.0–106.7	180	57.6 ± 3.2	45.7–67.0	180	19.2 ± 1.1	16.0–22.0
Female	174	13.0 ± 1.7	8.9–18.9	184	89.1 ± 4.6	77.0–104.0	185	55.1 ± 3.3	44.0–67.0	184	18.5 ± 1.1	16.4–22.5
Melanistic coyote	24	13.2 ± 2.0	7.8–15.8	22	87.8 ± 7.2	75.0–105.0	24	55.7 ± 5.5	43.2–68.7	24	18.9 ± 1.5	16.5–21.9
Male	13	12.9 ± 2.3	7.8–15.8	11	88.6 ± 6.4	75.0–96.5	13	56.0 ± 5.4	45.7–65.8	13	19.1 ± 1.6	16.5–21.6
Female	11	13.5 ± 1.6	11.4–15.5	11	87.0 ± 2.5	76.2–105.0	11	55.4 ± 5.9	43.2–68.7	11	18.6 ± 1.5	17.0–21.9
Gray hybrid	134	17.1 ± 4.2	6.4–27.5	137	98.1 ± 7.5	78.0–122.0	140	62.4 ± 4.5	50.3–79.9	140	20.5 ± 1.4	17.3–25.1
Male	79	17.6 ± 4.7	6.4–27.5	78	98.8 ± 7.9	78.0–122.0	81	63.0 ± 4.5	50.3–74.8	81	20.6 ± 1.4	17.3–25.1
Female	55	16.4 ± 3.5	7.3–23.2	59	97.2 ± 7.0	82.0–113.6	59	61.6 ± 4.5	52.0–79.9	59	20.2 ± 1.3	17.4–22.5
Melanistic hybrid	13	15.5 ± 2.7	10.2–18.5	13	93.4 ± 4.2	85.5–99.8	13	60.3 ± 3.0	53.0–65.3	13	19.9 ± 1.1	17.0–21.0
Male	4	17.1 ± 1.4	15.5–18.5	4	95.5 ± 5.3	88.0–99.8	4	62.9 ± 2.2	60.3–65.3	4	20.3 ± 0.5	19.6–20.7
Female	9	14.7 ± 2.8	10.2–18.5	9	92.5 ± 3.6	85.5–97.0	9	59.1 ± 2.6	53.0–62.1	9	19.7 ± 1.3	17.0–21.0

**Table 2:** Means (± SD) and ranges for head and appendage measurements of red wolves, coyotes, and hybrids in northeastern North Carolina, USA

*Canis* type	Head width (cm)			Ear length (cm)			Tail length (cm)		
	*n*	Mean	Range	*n*	Mean	Range	*n*	Mean	Range
Red wolf	180	11.9 ± 1.1	9.5–14.5	445	11.0 ± 0.6	9.0–12.9	443	36.4 ± 3.2	15.8–48.0
Male	89	12.3 ± 1.0	10.0–14.5	233	11.3 ± 0.6	9.0–12.9	232	37.3 ± 3.4	15.8–48.0
Female	91	11.5 ± 1.0	9.5–14.4	212	10.7 ± 0.5	9.3–12.5	211	35.5 ± 2.7	28.0–44.0
Gray coyote	270	10.8 ± 0.9	8.9–14.0	237	9.9 ± 0.6	8.0–12.8	239	33.8 ± 3.0	20.5–44.7
Male	135	11.1 ± 1.0	9.5–14.0	118	10.1 ± 0.6	8.7–12.5	118	34.2 ± 3.0	20.5–43.0
Female	135	10.5 ± 0.7	8.9–12.1	119	9.7 ± 0.6	8.0–12.8	121	33.4 ± 2.9	27.0–44.7
Melanistic coyote	15	10.7 ± 0.9	8.9–12.5	17	10.0 ± 0.9	9.0–12.5	16	34.7 ± 3.4	30.7–42.5
Male	11	10.9 ± 0.9	9.5–12.5	8	10.1 ± 0.6	9.3–11.2	7	34.7 ± 3.7	31.3–42.5
Female	4	10.2 ± 0.9	8.9–10.8	9	9.8 ± 1.1	9.0–12.5	9	9.8 ± 1.1	30.7–40.1
Gray hybrid	48	11.1 ± 0.8	78.0–122.0	140	10.5 ± 0.6	8.7–12.5	138	35.6 ± 3.1	24.5–43.5
Male	32	11.3 ± 0.6	9.5–12.5	81	10.7 ± 0.7	8.7–12.5	79	35.9 ± 3.3	24.5–43.5
Female	16	10.9 ± 1.0	9.5–12.5	59	10.4 ± 0.6	9.2–11.4	59	35.2 ± 2.8	27.0–41.5
Melanistic hybrid	2	10.5 ± 1.4	9.5–11.5	13	10.1 ± 0.6	8.5–10.9	13	36.2 ± 2.7	31.5–40.0
Male	N/A	N/A	N/A	4	10.6 ± 0.3	10.2–10.9	4	37.3 ± 2.0	34.5–39.0
Female	2	10.5 ± 1.4	9.5–11.5	9	9.9 ± 0.6	8.5–10.4	9	35.7 ± 3.0	31.5–40.0

**Fig. 3:**
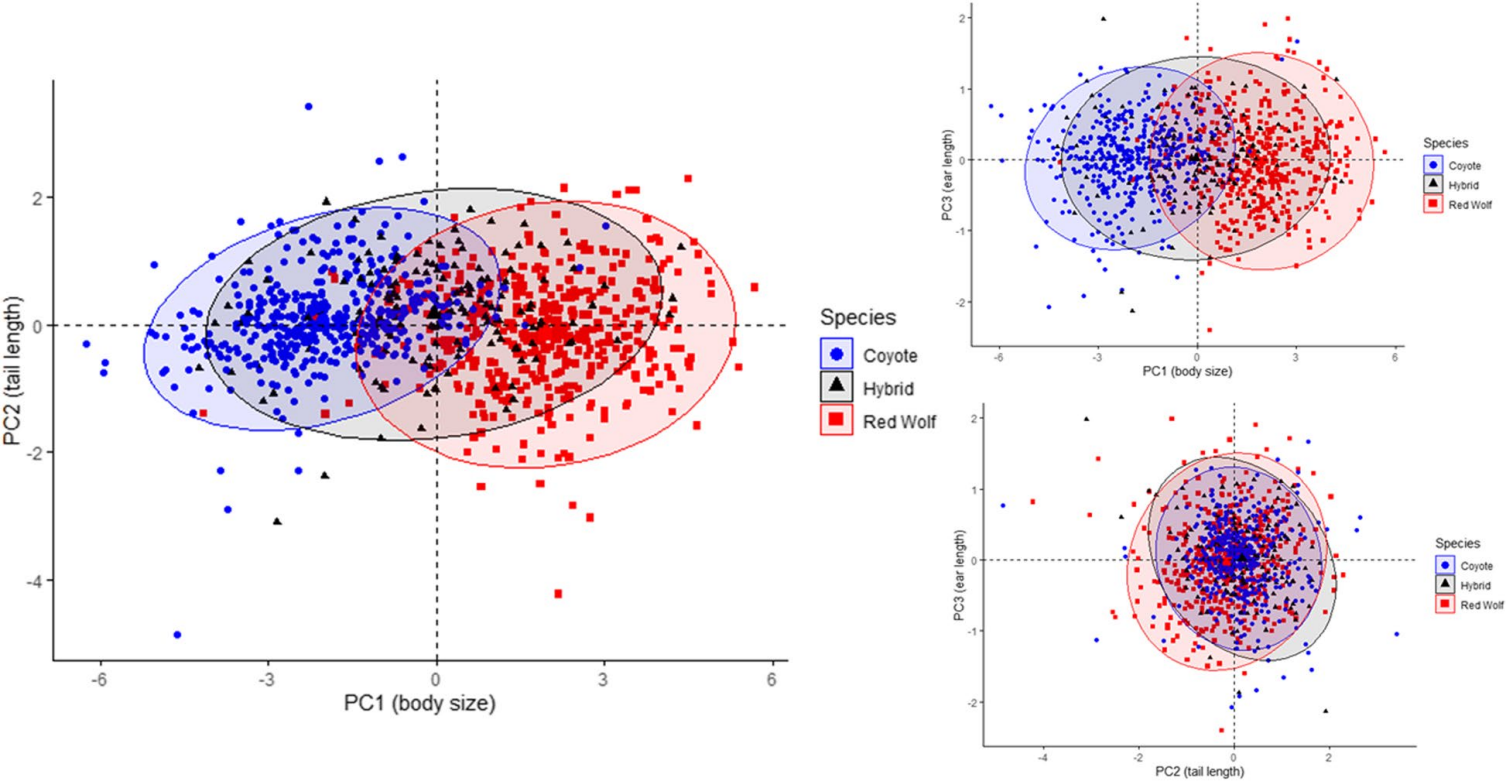
Scatter plots of component loadings for between-group principal components of the principal component analysis. Shaded circles represent 95% ellipses

For our analysis, we used canids with > 4 of the 6 body measurements recorded. We assessed the measurements of 425 coyotes, 449 red wolves, and 153 hybrids using principal component analysis (PCA) (Fig. 3). Approximately 31.8, 37.6, and 30.1% of the data sets were complete for coyotes, red wolves, and hybrids, respectively. Width of head was the most commonly missing body measurement (46.5% of individuals) followed by ear (17.5%), tail (17.2%), body mass (4.1%), body length (1.4%), shoulder height (1.1%), and hind foot length (0.3%).

**Fig. 4:**
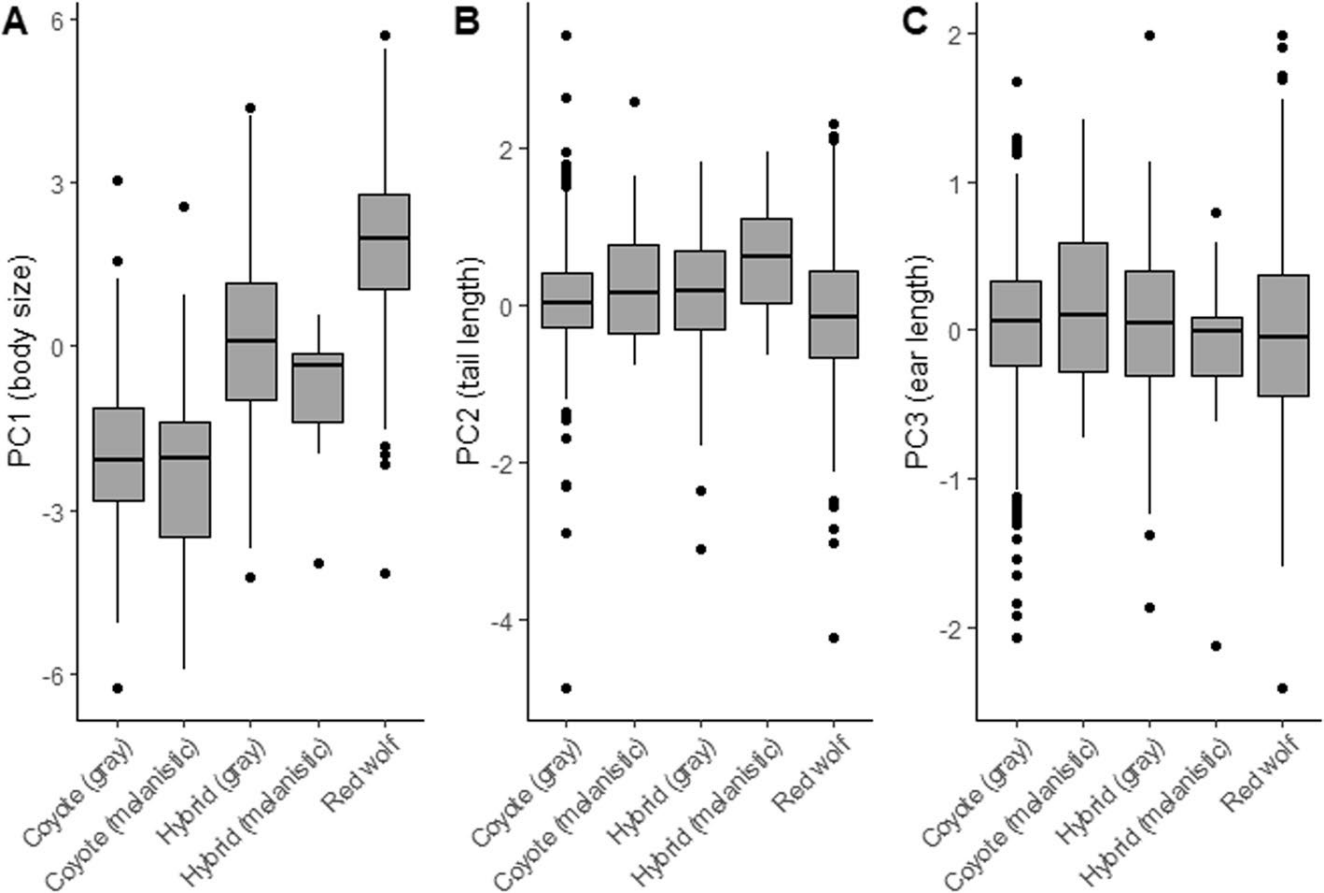
Mean principal components scores for (**A**) PC1 (body size), (**B**) PC2 (tail length), and (**C**) PC3 (ear length) of coyotes, red wolves, and hybrids in the southeastern United States

Only principal component (PC) 1, which explained 75.84% of the cumulative variation, had an eigenvalue > 1 (Table 3). The eigenvalues of PC1 consisted of strong positive loadings for all body measurements. Although the eigenvalues for PC2 and PC3 were < 1, both contributed 14.0% of the cumulative variance explained in our PCA (Table 3) and contained loadings of body measurements that were independent of body size. Collectively, these PC scores indicated that once PC1 accounted for body size, PC2 and PC3 accounted for variation in tail length and ear length, respectively. Mean PC1 (body size) scores for hybrids were intermediate to those for coyotes and red wolves (*F*_*4*, *1021*_ = 449.300, *P* < 0.001; Fig. 4). We observed differences in mean PC2 (tail length) scores (*F*_*4*, *1021*_ = 8.729, *P* < 0.001; Fig. 4) which indicated red wolves had shorter tails relative to their body size than did coyotes and hybrids, although mean tail length of red wolves was greater than mean tail length of coyotes and hybrids (Table 2). We detected no differences in mean PC3 (ear length) scores (*F*_*4*, *1021*_ = 1.098, *P* = 0.356; Fig. 4), although mean ear length of red wolves was greater than mean ear length of coyotes and hybrids (Table 2). We detected no difference in mean PC scores of melanistic and gray coyotes and mean PC scores of melanistic and gray hybrids (Fig. 4). In other words, we found no evidence that melanism was correlated with differences in morphometrics observed within our *Canis* taxa.

**Table 3:** Factor loadings and percent contribution of body measurements for each of the top 3 principal components for body measurements recorded from red wolves, coyotes, and their hybrids across southeastern United States. Also included are eigenvalue, percent of total variance explained, and descriptions of principal components

Body measurements	Principal component 1		Principal component 2		Principal component 3	
	Loading	% Contribution	Loading	% Contribution	Loading	% Contribution
Body mass	0.94	16.57	−0.12	2.16	−0.09	2.39
Ear length	0.85	13.74	−0.03	0.14	0.50	73.69
Tail length	0.64	7.65	0.75	88.88	−0.06	0.96
Body length	0.92	15.83	−0.11	1.91	−0.18	9.60
Hind foot length	0.92	15.89	−0.05	0.46	0.10	3.09
Shoulder height	0.93	16.17	−0.03	0.17	−0.12	4.20
Head width	0.87	14.14	−0.20	6.29	−0.14	6.07
Eigenvalue	5.30		0.64		0.33	
% of total variance	75.77		9.14		4.76	
Description	Body size		Tail length		Ear length

### Space use and habitat selection

**Fig. 5:**
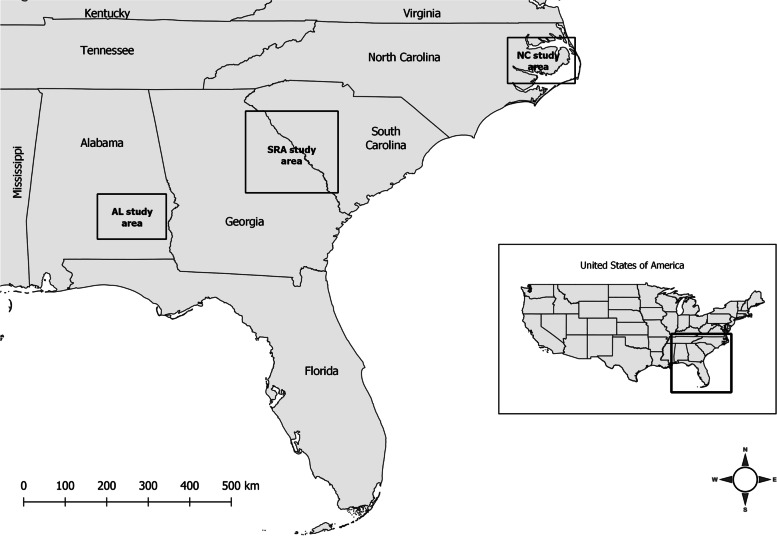
Map of the three study areas (indicated by boxes) in the southeastern United States. The Savannah River Area (SRA) represented the Georgia and South Carolina study areas

To assess space use and habitat selection, we monitored 6 clusters of coyotes, each cluster consisting of 5 animals fitted with GPS radio-collars (*n* = 30 coyotes, Table 4). Our 6 clusters consisted of 20 resident (14 gray, 6 melanistic) and 10 transient (7 gray, 3 melanistic) coyotes. We included random intercepts for each coyote nested within clusters in our generalized linear mixed models (GLMMs) to account for unbalanced telemetry data and differences in land cover among 3 geographic regions (Fig. 5) when comparing space use and habitat selection by melanistic coyotes to selection by gray coyotes. Our GLMMs indicated that gray coyotes maintained smaller home ranges than did melanistic coyotes (*β* = − 17.432 ± 7.188 SE, *P* = 0.015), whereas no difference was observed between the size of core areas (*β* = − 1.518 ± 1.932 SE, *P* = 0.432), transient ranges (*β* = 191.9 ± 184.0 SE, *P* = 0.297), and biding areas (*β* = 20.86 ± 32.95 SE, *P* = 0.527) of gray and melanistic coyotes (Table 4). Mean home range sizes for gray and melanistic coyotes were 17.4 km^2^ (SD = 10.6) and 27.1 km^2^ (SD = 12.7), respectively. Mean core area size for coyotes was 4.0 km^2^ (SD = 2.3), whereas mean sizes for transient ranges and biding areas were 179.9 km^2^ (SD = 138.0) and 22.7 km^2^ (SD = 22.1), respectively.

**Table 4:** Summary of coyote collar deployments, geographical clusters, color, space use status, monitoring dates, and extent of space used (95 and 50% contour intervals representing home ranges and core areas, respectively for residents and transient ranges and biding areas, respectively for transients) in the southeastern United States. The “M” of “F” in coyote ID indicates sex

Cluster	Coyote ID	Color	Status	Duration monitored (days)	95% (km^2^)	50% (km^2^)
Alabama	AL08F	Gray	Resident	498	8.5	1.5
	AL14M	Gray	Resident	389	26.1	5.2
	AL17F	Gray	Resident	281	10.2	2.3
	AL19F	Gray	Resident	323	14.2	2.4
	AL23M	Melanistic	Resident	198	36.1	6.6
Georgia I	GA03F	Gray	Resident	231	12.0	3.0
	GA08F	Gray	Resident	538	12.6	7.6
	GA19F	Melanistic	Resident	497	17.3	10.2
	GA22M	Gray	Resident	280	10.6	4.9
	GA25M	Melanistic	Resident	358	17.7	2.2
Georgia II	GA28F	Gray	Transient	121	40.6	5.3
	GA37F	Gray	Transient	285	170.9	20.8
	GA38M	Gray	Transient	132	231.5	43.4
	GA40M	Melanistic	Transient	225	132.0	13.9
	GA42M	Gray	Transient	135	50.1	11.0
South Carolina I	SC27M	Gray	Resident	482	13.0	2.2
	SC33F	Gray	Resident	441	8.3	1.2
	SC34M	Melanistic	Resident	338	15.2	3.4
	SC41F	Gray	Resident	469	16.8	3.3
	SC45M	Gray	Resident	368	12.9	3.0
South Carolina II	SC29F	Gray	Transient	321	204.5	17.3
	SC30M	Melanistic	Transient	438	207.3	18.0
	SC32M	Melanistic	Transient	295	121.8	10.7
	SC36F	Gray	Transient	355	72.9	8.8
	SC37M	Gray	Transient	225	517.7	78.1
North Carolina	20443F	Gray	Resident	419	25.5	2.6
	20503F	Gray	Resident	368	47.3	7.2
	20,566 M	Gray	Resident	463	25.9	3.3
	40,458 M	Melanistic	Resident	146	46.8	4.4
	40,586 M	Melanistic	Resident	140	29.7	4.3

We detected differences in habitat selection by gray and melanistic coyotes (Tables 5 and 6) and our Spearman’s rank correlations from *k*-fold cross-validation indicated that our best models consistently predicted habitat selection patterns for gray (*r*_s_ = 0.814, *P* < 0.001) and melanistic (*r*_s_ = 0.813, *P* < 0.001) coyotes.

**Table 5:** Summary of top generalized linear mixed models (cumulative Akaike weight ≤ 1.00) for predicting 3rd-order habitat selection by melanistic and gray coyotes in the southeastern United States. Shown are Akaike’s Information Criteria for small sample sizes (AICc), differences among AICc (ΔAIC), AICc weights (ω_i_) and cumulative AICc weights

*Canis* type	Model	*k*	AIC_c_	ΔAIC_c_	ω_i_	Cumulative ω
Gray coyote	CA^1^ + RD^2^ + SH^3^ + HD^4^ + WL^5^+ AG^6^	9	188,761.70	0.00	0.69	0.69
	Full model	10	188,763.40	1.69	0.29	0.98
	CA + SH + HD + WL + AG	8	188,767.80	6.04	0.01	0.99
	CA + FO^7^ + SH + HD + WL+ AG	9	188,769.40	7.69	0.01	1.00
	AG + CA + RD + SH + WT	8	188,783.50	21.82	0.00	1.00
Melanistic coyote	Full model	10	63,358.12	0.00	0.35	0.35
	AG + CA + FO + RD + HD + WT	9	63,358.92	0.80	0.23	0.58
	CA + FO + RD + HD + WT	8	63,359.10	0.98	0.21	0.79
	CA + FO + RD + SH + HD + WT	9	63,359.32	1.20	0.19	0.98
	AG + CA + FO + SH + HD + WT	9	63,336.00	7.88	0.01	0.99

^1^% canopy cover, ^2^distance to roads, ^3^distance to shrubland, ^4^distance to human development, ^5^distance to wetlands, ^6^distance to agriculture, ^7^distance to forests

**Table 6:** Model averaged parameter estimates for 3rd-order resource selection functions for radio-collared coyotes in the southeastern United States. Shown are β coefficients, standard error (SE), and 95% confidence intervals (CI)

Coat color	Model variables	β	SE	95% CI
Gray coyote	Intercept	−1.115	0.062	−1.239, −0.992
	Percent canopy cover	0.054	0.007	0.039, 0.066
	Distance to agriculture	−0.257	0.008	−0.273, −0.240
	Distance to forest	−0.002	0.005	−0.023, 0.013
	Distance to roads	0.037	0.016	0.012, 0.066
	Distance to development	−0.037	0.008	−0.052, −0.022
	Distance to shrubland	−0.087	0.008	−0.103, −0.071
	Distance to wetlands	0.148	0.007	0.134, 0.162
Melanistic coyote	Intercept	−1.112	0.028	−1.167, − 1.056
	Percent canopy cover	0.238	0.012	0.213, 0.259
	Distance to agriculture	0.012	0.014	−0.002, 0.047
	Distance to forest	0.090	0.014	0.068, 0.120
	Distance to roads	−0.048	0.017	−0.080, − 0.018
	Distance to development	−0.045	0.012	−0.069, − 0.024
	Distance to shrubland	−0.011	0.014	−0.046, 0.004
	Distance to wetlands	−0.063	0.012	−0.087, − 0.038

**Fig. 6:**
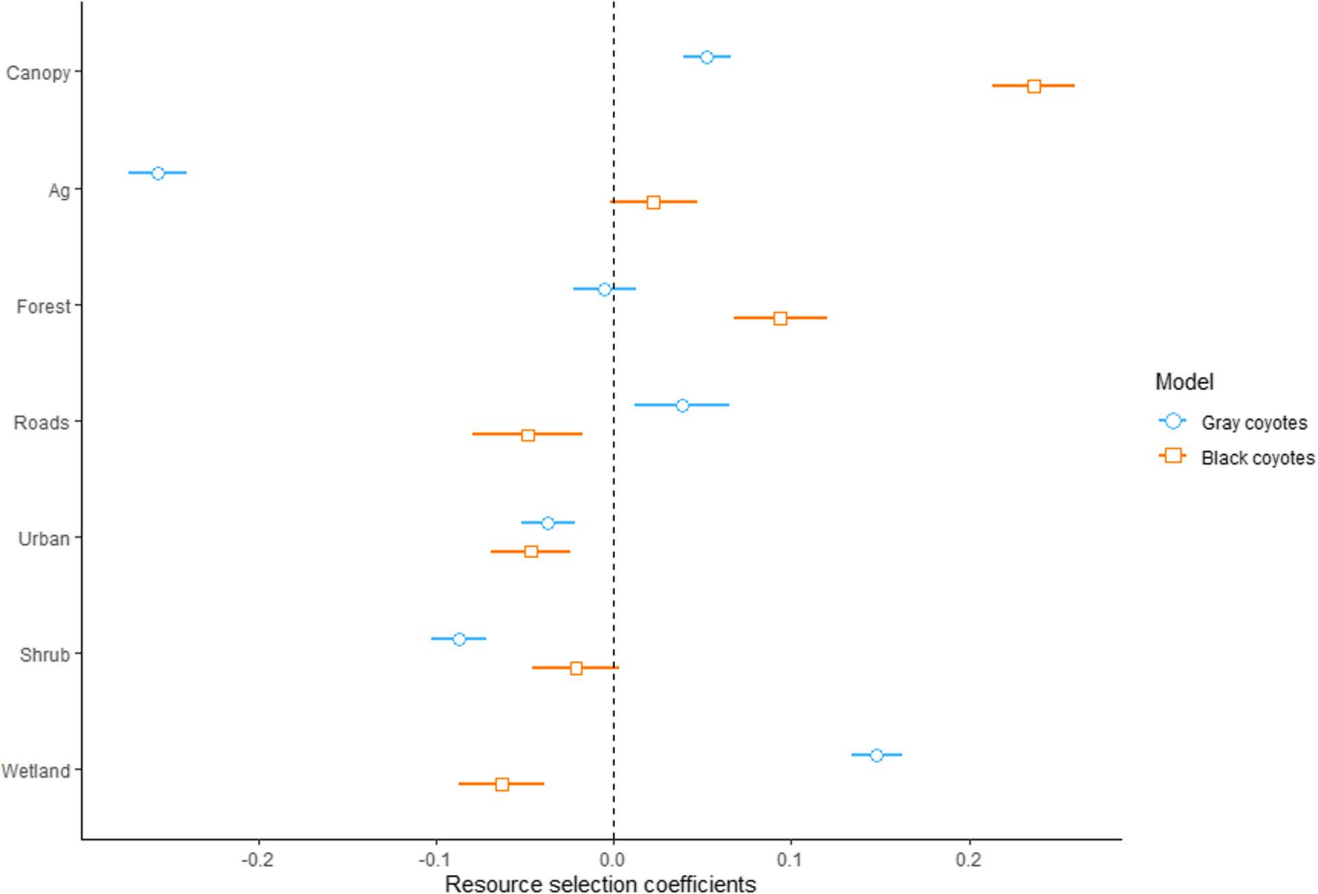
Model averaged parameter estimates for 3rd-order resource selection functions for radio-collared melanistic and gray coyotes in the southeastern United States during 2009–2017. The midpoint location and 95% confidence intervals of each distribution are represented by shapes and lines, respectively. Canopy = % canopy cover, Ag = distance to agriculture, Forest = distance to forest, Roads = distance to roads, Urban = distance to human development, Shrub = distance to shrubland, and Wetland = distance to wetland

For gray coyotes, all land cover types except for forest were important predictors of habitat selection (Table 5). Gray coyotes exhibited strong selection for agriculture and strong avoidance of wetlands (Table 6 and Fig. 6). They also showed selection for areas in or proximate to shrubland and human development with increasing canopy cover. Gray coyotes avoided roads. For melanistic coyotes, all landcover types except for agriculture and shrubland were important predictors of habitat selection (Table 5). Melanistic coyotes exhibited strong selection for areas with increasing canopy cover (Table 6 and Fig. 6). They also showed selection for areas in or proximate to wetlands, human development, and roads and avoided areas with forest cover.

### Survival

We documented 256 mortalities of radio-collared coyotes (64.1% of mortalities) and hybrids (35.9% of mortalities) from the Red Wolf Experimental Population Area (hereafter NC Recovery Area) in northeastern North Carolina during 1992–2018 in which melanistic animals accounted for 7 mortalities. Global tests suggested that our data did not violate the proportional hazards assumption (maximum *χ*^2^ = 0.322, *P* = 0.85) in which the relative hazard between coyotes and hybrids or gray and black phenotypes was constant over time and did not cause survival curves to diverge. Mean annual survival did not differ between coyotes (0.673 [0.629–0.720 95% CI]) and hybrids (0.689 [0.630–0.754 95% CI]; *z* = 0.357, *P* = 0.721, hazard = 1.062 [0.765, 1.474 95% CI]). However, melanistic animals exhibited greater annual survival than did gray conspecifics (0.827 [0.716–0.955 95% CI] vs 0.671 [0.639–0.705 95% CI]; *z* = − 1.892, *P* = 0.059, hazard = 0.478 [0.222, 1.027]).

## Discussion

Although Anderson et al. [9] postulated that melanism in North American gray wolves and coyotes was introduced through hybridization with domestic dogs, Rutledge et al. [30] suggested that a more comprehensive examination of black canids from eastern North America was required before such conclusions of introgressive hybridization from dogs to gray wolves and coyotes were drawn. We agree with Rutledge et al. [30] because melanism was historically common in red wolves [21, 26, 32, 40, 41] and absent from coyotes [31]. Coyotes that colonized the Southeast during the twentieth century likely acquired melanism through hybridization with red wolves, given that melanistic coyotes were reported soon after they made inroads into the region following the decline of red wolves [21, 32] and occur predominantly in populations inhabiting the red wolf’s historical range.

**Fig. 7:**
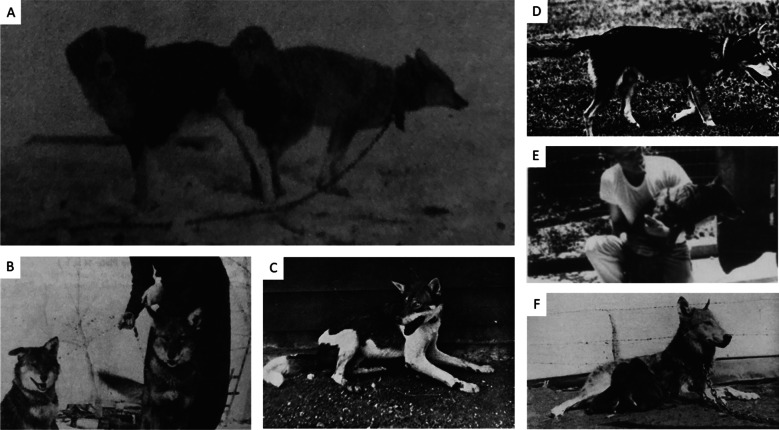
**A** A captive western coyote copulating with a collie-like domestic dog [32]. **B** Two captive coyote-dog hybrids in Wyoming, USA [32]. **C** A captive red wolf-dog hybrid from Reynolds County, Missouri, USA [43]. **D** An F_2_ coyote-beagle hybrid who was part of a scientific study conducted in St. Louis, Missouri, USA [43]. **E** An F_1_ coyote-dog hybrid who was part of a scientific study from Lawrence, Kansas, USA [44]. **F** A captive female gray wolf nursing her litter of hybrid pups in Hall County, Texas, USA [44]. Her pups were the result of humans crossing her with a hound dog

Regarding dog introgression that co-occurs with melanism in southeastern coyote populations, the origin of that introgression may have occurred through human-facilitated hybridization in captive settings by which some hybrid escapees backcrossed with wild coyotes or hybridization with red wolves in the wild who may have carried dog alleles. Indeed, Mengel [42] reported that some of his F1 and F2 coyote-dog hybrids escaped from their pens and were not retrieved and Goldman [21] reported the existence of a red wolf-dog hybrid in Reynolds County, Missouri (Fig. 7c) indicating that humans crossed red wolves and dogs in captivity. In fact, there is considerable evidence of human-facilitated interbreeding of wolves, coyotes, and dogs in captive environments (Fig. 7) [21, 31, 42–45], whereas direct interbreeding (e.g., copulation; Fig. 7a) between coyote and dogs has not been documented in the wild (see review by vonHoldt and Aardema [46]) despite numerous research and monitoring programs and the common and widespread use of camera surveys in modern research. However, interbreeding between coyotes and red wolves in the wild has been repeatedly and comprehensively documented by ecological [47–50] and molecular [51–53] studies by which more realistic and parsimonious pathways (e.g., red wolf and coyote hybridization) can be formulated for how southeastern coyotes may have acquired melanistic traits and dog alleles.

Melanistic coyotes and hybrids comprised 2.0–8.5% of individuals captured at our study areas indicating that the occurrence of melanism was relatively low but consistent in the region’s coyote populations. This observation corroborates previous studies reporting that melanistic individuals comprised < 10% of coyote populations surveyed in other parts of the Southeast [22, 25]. Few studies on the occurrence of melanism in red wolves exist, and the actual proportion of the historical wolf population that consisted of melanistic individuals is unknown. However, anecdotal accounts and population surveys suggest that melanistic individuals were more common in the historical red wolf population than they are in today’s coyote population. For example, the only three pictures of free roaming wild red wolves in Louisiana were photographs of melanistic wolves captured by remote cameras [40], and red wolves were often referred to as the “black timber wolf” by Louisiana zoologists [26]. During the same period of Gregory’s [40] photographic expedition of northeastern Louisiana, a survey of red wolves in Arkansas reported that 25% of the population consisted of melanistic wolves [41]. Elder and Hayden [54] reported that five of seven Missouri specimens considered to be red wolves or red wolf-coyote hybrids were melanistic. Therefore, we suggest that melanism in the historical red wolf population may have occurred at similar frequencies as those observed in gray wolves inhabiting boreal forests of North America [8–10, 16] and occurred more frequently in historical red wolves than in contemporary coyotes of the Southeast.

We observed no correlation between *Canis* morphometrics and pelage color, as melanistic coyotes and hybrids were similar in size as their gray conspecifics. Instead, body size of canids was driven by red wolf ancestry in which red wolves represented a uniquely large *Canis* phenotype that was not achieved by coyotes and hybrids [49]. Our findings offer evidence that the tails and ears of red wolves and coyotes are distinctive characteristics [49]. Our interpretation of PC2 (tail length) and PC3 (ear length) suggests that the distinctiveness was minor, only meaningful when related to their body sizes, and consisted of red wolves having shorter and less bushier tails than did coyotes. Additionally, the red wolf’s triangular facial appearance is accentuated by the angle at which the wolf carries its ears [47]. Despite efforts to quantify coyote niche expansion through hybridization in the northeastern United States and eastern Canada [36, 55–57], no field research has substantiated the assumption that intermediate body sizes facilitate unique niches for introgressed coyote populations in the Southeast. To our knowledge, coyote populations with the greatest levels of red wolf ancestry are largely restricted to the NC Recovery Area of northeastern North Carolina [58] and isolated pockets along the American Gulf Coast [59–61] indicating reduced niche dimensions when compared to other southeastern coyote populations with less wolf ancestry. Furthermore, we detected no differences in annual survival rates for coyotes and hybrids in the NC Recovery Area. We suggest that future research directly study the effects of red wolf ancestry in *Canis* populations of the region to confirm claims that hybridization can confer adaptive benefits to *Canis* taxa.

Mean home-range size of melanistic coyotes was 1.6 times larger than that observed for gray coyotes, and melanistic coyotes exhibited stronger selection for areas with canopy and wetland cover than did gray coyotes. Research on space use of coyotes and red wolves in the NC Recovery Area reported that body size influenced home range sizes and that coyote home range size was negatively correlated with agriculture [50, 62]. Given that we detected no difference in body sizes of melanistic and gray coyotes, it is likely that differences in land cover preferences by coyotes influenced their home range sizes such as gray coyotes exhibiting stronger selection for agriculture than did melanistic coyotes. Wetlands in our study areas were predominantly woody wetlands characterized by coastal bottomland forest and other types of woody riparian cover [62, 63]. Therefore, forest cover that melanistic coyotes avoided were drier deciduous, evergreen, and mixed forests that gray coyotes used according to their availability. Regardless of pelage color, coyotes exhibited selection for areas impacted by human development; however, gray coyotes showed avoidance of roads whereas melanistic coyotes exhibited selection for them. We speculate that the selection of roads by melanistic coyotes likely resulted from their need to use linear corridors to maneuver around inundated areas associated with woody wetland cover whereas gray coyotes could avoid roads as they selected for drier, open habitats that allowed for more diffuse movements. Nevertheless, our findings corroborate observations and insights spanning from 18th- and 19th-century naturalists to modern biologists, who linked the occurrence of melanism in wild *Canis* populations of the Southeast with dense canopy cover [19–23, 25] and support hypotheses such as Gloger’s rule that postulate an adaptive role for melanism in canopy dense environments [7, 35, 64].

Melanistic coyotes can be cryptic under dense canopy cover in which their black pelage may improve anti-predator (i.e., human hunters) behaviors through superior camouflage [35, 64]. For example, melanistic coyotes and hybrids in the NC Recovery Area exhibited greater survival than did their gray counterparts, which may be attributed to better concealment in coastal bottomland forests. We suggest that the large home range sizes of melanistic coyotes was caused by their preferences for areas with dense canopy, and their selection for lower quality wetland cover was likely a trade-off of reduced foraging efficiency for improved survival. Large home ranges and differential use of land cover by melanistic coyotes may facilitate weak assortative mating in eastern coyote populations, whereby melanistic animals have lower success of finding compatible mates in comparison to their gray conspecifics. Indeed, space use behaviors influenced assortative mating in red wolves and coyotes [50] and melanistic coyotes selected for similar land cover types (i.e., wetland cover) as did red wolves in eastern North Carolina [65]. Furthermore, Hinton et al. [29] suggested that red wolf ancestry improved coyote dispersal capabilities, rather than their ability to kill deer [36, 38, 66], improving connectivity among coyote metapopulations in a region dominated by forest cover. Given that melanism is a newly acquired trait in eastern coyote populations that may influence coyote space use behaviors, we believe some reproductive isolation between gray and melanistic individuals along the colonization front may have been important for engendering diversity and dispersal of newly acquired traits.

## Methods

### Study area

We compiled data from 3 regions in the Southeast representing 3 separate *Canis* populations: North Carolina’s Albemarle Peninsula, the Savannah River area along the Georgia and South Carolina border, and southeastern Alabama (Fig. 5). Since 1987, the Albemarle Peninsula of northeastern North Carolina served as the NC Recovery Area [28]. The peninsula included 5 counties (Beaufort, Dare, Hyde, Tyrrell, and Washington) and consisted of approximately 6000 km^2^ of federal, state, and private lands. The NC Recovery Area was predominantly an intensively farmed agricultural-hardwood bottomland mosaic in which approximately 30% of the landscape was croplands. More details of the NC Recovery Area can be found in Hinton et al. [67].

During 2015–2016, we captured and monitored coyotes across several broad areas in southeastern Alabama (Barbour, Macon, and Pike Counties), east-central Georgia (Columbia, Jefferson, Lincoln, McDuffie, and Warren Counties), and western South Carolina (Aiken, Edgefield, McCormick, and Saluda Counties) totaling approximately 16,200 km^2^ (Fig. 5). Because some coyotes captured at the Georgia and South Carolina study areas dispersed into each respective study area, we considered coyotes in both areas along the Savannah River to be one population and referred to the area as the Savannah River area (SRA). Land cover in Alabama and SRA contained a mix of early successional, agricultural, forested, and urban habitats. More details on these two study areas can be found in Ward et al. [63].

### Animal captures

Red wolves, coyotes, and hybrids were captured with foothold traps with offset jaws (Victor #1.5 and #3 softcatch, Woodstream Corporation, Lititz, Pennsylvania, USA, and Minnesota Brand 550, Minnesota Trapline Products, Pennock, Minnesota, USA). From 1987 to 2011, the Recovery Program captured red wolves, coyotes, and hybrids in northeastern North Carolina. During 2015–2016, the University of Georgia captured coyotes in Alabama and SRA.

Once captured, animals were restrained with a catchpole, muzzle, and hobbles. However, we chemically immobilized some animals with an intramuscular injection of 1.3–1.8 mg/kg ketamine HCl and 0.2–0.4 mg/kg xylazine HCl to inspect inside their mouths for injuries. Sex, weight, body measurements, and pelage color were recorded for all animals, and ages of coyotes and hybrids were estimated by tooth wear [68, 69]. We acquired accurate estimates of red wolf ages through the Recovery Program’s detailed life-history data [49, 70]. Ancestry of all animals was confirmed with microsatellite markers and genomic information [29, 49, 71, 72]. We categorized animals ≥2 years as adults, 1–2 years old as juveniles, and < 1 year old as pups. Our capture and handling of animals followed guidelines approved by the American Society of Mammalogists [73] and were approved by the Institutional Animal Care and Use Committees at the University of Georgia (A2014 08–025-R2) and Louisiana State University (AE2009–19).

Prior to release at their capture sites, all red wolves, coyotes, and hybrids were fitted with mortality-sensitive radio-collars. In the NC Recovery Area, animals were predominantly fitted with very high frequency (VHF) radio collars (Telonics, Inc., Mesa, AZ) for monitoring space use, breeding status, and survival [70]. During 2005–2011, some red wolves and coyotes were fitted with global positioning system (GPS) radio collars for research purposes [62, 65, 74, 75]. Red wolves equipped with GPS radio-collars (Lotek 4400S, Newmarket, Ontario, Canada) had their locations recorded every 5 hours on a 24-hour rotating schedule throughout the year. Coyotes in the NC Recovery Area equipped with smaller Lotek 3300 s GPS collars had their locations recorded every 4 hours (e.g., 0000, 0400, 0800, 1200, and so on). The Recovery Program monitored radio-collared red wolves, coyotes, and hybrids 2–3 times a week from aircraft to identify and monitor wolf territories in the NC Recovery Area. Coyotes in Alabama and SRA fitted with G2110E satellite GPS collars (Iridium; Advanced Telemetry Systems, Isanti, Minnesota, USA) had their locations recorded every 4 hours beginning at midnight.

### Morphometrics

We recorded postcranial measurements including body length (anterior tip of the nose pad to the tail base), tail length (tip of the fleshy part of the tail to the tail base), hind foot length (hock to the tip of the digital pads), and shoulder height (tip of the scapula to tip of the digital pads). Cranial measurements included width of head (most widely separated points) and ear length (edge of the external auditory canal to the tip of the ear).

We used a PCA to extract the dominant, underlying gradients of variation (principal components) in our dataset [76]. The PCs are weighted linear combinations of the original variables ordered according to the amount of variation each PC explained. We logarithmically transformed our data, as body mass was measured on a different scale than linear body measurements.

Approximately 66% of our study animals were missing at least 1 body measurement which makes deletion of individuals or traits from the analysis impractical. In this context, we addressed the issue of missing values within our morphometrical dataset by using a joint modeling approach in the R package Amelia [77] to create a completed dataset to perform the PCA. The joint modeling approach obtains maximum likelihood estimates using an expectation-maximization algorithm and considers the relationships between variables to fill the gaps [78]. In doing so, the joint modeling approach allowed for missing value uncertainty to be incorporated into our PCA [78]. We used the relative percent variance criterion jointly with the latent root criterion (PCs with eigenvalues > 1) to determine the number of significant PCs to retain and interpret, because the latent root criterion is known to be overly conservative when the number of variables is < 20 [79]. We then based our interpretation of each PC on those variables with loadings ≥0.50 or ≤ − 0.50 and placed most emphasis on those with loadings ≥0.60 or ≤ − 0.60 [79]. We used variables with the strongest loadings to interpret the ecological meaning of each PC.

### Space use and habitat selection

We used only GPS-collared coyotes for our space use and habitat selection analyses for two reasons. First, red wolves could not be included in this analysis because melanistic individuals were absent in the extant wolf population. Additionally, estimates of wolf space use and habitat selection have been reported [65, 75, 80]. Second, we excluded coyotes and hybrids in the NC Recovery Area who were fitted with VHF radio-collars because they were not monitored intensively to have achieved sufficient numbers of locations (e.g., ≥30) required for reliable estimates of home range size and habitat selection [81].

To identify resident coyotes, we used an animal’s spatial association with other animals and fidelity to an area for ≥4 months as our primary criteria [62, 63]. We confirmed the presence of mates and pack members through field inspection for sign (i.e., visual observation and tracks) of other individuals over the course of several weeks [62, 63]. Wide-ranging and unstable space use by animals was characteristic of transient behaviors, as these individuals were typically young dispersers that moved nomadically on the landscape in search of mates and territories and, therefore, did not maintain home ranges [62]. Accordingly, we did not refer to transient space use as home ranges and core areas, but instead refer to space used by transient coyotes as transient ranges and biding areas [62]. Transient ranges and biding areas are analogous to home ranges and core areas, but are assigned to non-breeding, solitary animals traversing the landscape seeking mates and territories whereas home ranges and core areas were assigned to animals belonging to breeding pairs and packs who defended territories.

To investigate space use and habitat selection by coyotes, we identified local clusters of GPS-collared coyotes that included a melanistic individual (Table 1). This approach allowed us to account for differences in land cover available to coyotes inhabiting the 3 geographic areas when comparing habitat selection by melanistic coyotes and gray coyotes. Using the locations of our GPS-collared melanistic coyotes, we identified 6 unique clusters of coyotes across our Alabama, SRA, and North Carolina study areas where melanistic individuals resided. We created clusters of 5 coyotes by identifying the social status of melanistic coyotes (resident vs. transient) and assigned the 3–4 closest gray coyotes of the same social status to the cluster. For example, in Alabama, we captured and GPS-collared 54 coyotes between 2015 and 2016. Of those 54 coyotes, only 1 coyote was melanistic. We used that coyote to create the Alabama cluster, and because that melanistic coyote was a resident animal, we selected the 4 closest resident gray coyotes to create the Alabama cluster (Table 1).

We estimated space use of resident and transient coyotes using dynamic Brownian bridge movement models (dBBMM) in Program R 3.6.3, using the package move [82, 83]. For full tracks of each animal, we measured variation in movements using a moving window size of 7 locations (equivalent to 14 hours) with a margin of 3 locations while assuming an error estimate of 20 m for all locations [62]. For resident coyotes, we defined 95 and 50% contour intervals as home ranges and core areas, respectively [62]. For transient coyotes, we defined 95 and 50% contour intervals as transient ranges and biding areas, respectively [62]. To account for effects of pelage color on coyote space use, we used generalized linear mixed models (GLMM) with a logit link in Program R [84]. Our response variables were home range and core area sizes for resident animals, and transient range and biding area sizes for transients. We modeled pelage color as a binary (0 = gray, 1 = melanistic) predictor variable and included random intercepts for each coyote nested within clusters to account for the influence of unbalanced telemetry data and unmeasured geographic-related factors.

To develop resource selection functions (RSF), we followed a 3rd-order resource selection design [85] to examine the relationship between land cover and coyote space use within 95% ranges. We used individual coyotes as sampling units and measured resource availability for each animal at the 95% contour intervals estimated via dBBMMs. To estimate RSFs, we used a binomial approach by comparing characteristics of known locations to 3 times the number of random locations within 95% contour intervals for each coyote [85]. We generated 3 times more random locations than GPS locations for each coyote to ensure accurate estimates of land cover availability for use with RSFs [86].

We overlaid GPS locations and random locations onto 30-m resolution digital maps of 7 measurements of major land cover types (percent canopy cover, distance to roads, distance to agriculture, distance to forest, distance to shrubland, distance to human development, and distance to wetlands) that likely influenced coyote habitat selection. Using the Euclidean Distance tool in the Spatial Analyst toolbox in ArcGIS 10.7 (Environmental Systems Research Institute Inc., Redlands, California), we calculated distances from every 30-m pixel to the closest landscape features (agriculture, forest, shrubland, human development, and wetlands) using the United States Geological Survey (USGS), National Land Cover Database from 2011 and 2016 [87, 88]. Our forest cover category was created by reclassifying the NLCD’s deciduous, evergreen, and mixed forest land covers as “forest” cover. We acquired roads layers from the Alabama, Georgia, and North Carolina Departments of Transportation to create a distance to roads layer. Finally, we used percent canopy cover derived from the 2011 and 2016 NLCD tree canopy cover layers.

We used GLMMs with a logit link in Program R to evaluate 3rd-order selection by coyotes [84, 85, 89]. We included random intercepts for each coyote nested within clusters in each model using the lme4 package in R [90]. Including random intercepts for individual coyotes and clusters accounted for the influence of unbalanced telemetry data and unmeasured geographic-related factors. We modeled resource selection with a binary (0 = random, 1 = known) response variable. Before modeling, we rescaled and centered values for distance-based variables and canopy cover by subtracting their mean and dividing by 1 standard deviation. To model the influence of land cover correlates on the relative probability of habitat selection by melanistic and gray coyotes, we constructed separate but identical GLMMs for each coyote type and compared the coefficients and their 95% confidence intervals [86]. As noted by Northrup et al. [86], non-spatial factors such as pelage color or sex cannot be modeled as covariates in spatial models because these models are typically approximating a Poisson point process model.

We then restricted models to first-order terms and explored all possible subsets of the 7 predictors including the null model as candidate models to investigate coyote habitat selection. We evaluated model sets using Akaike’s Information Criterion adjusted for small sample sizes (AICc) and used ΔAICc to select which models best supported factors influencing resource selection by coyotes [91]. We considered the model with the lowest AICc and the greatest model weight as the best approximating model. However, when model sets had ≥2 models that were within 2 ΔAICc of the top model, we performed model-averaging across the top model set to calculate effect sizes of explanatory variables. We only considered parameter estimates with 95% confidence intervals that excluded 0 to be informative.

We then validated our top models describing coyote habitat selection influenced by pelage color using k-fold (k = 5 repetitions) procedures to assess the predictive performance of RSF models by randomly dividing our data into 5 equally sized folds and using 4 folds to create our training data set (80% of the data) and the last fold (20% of the data) as our test data set. We classified RSF values into 5 quantile bins and calculated each bin from the test data set. We used Spearman’s rank correlation to compare expected and observed frequencies [92]. Models with good predictive abilities are expected to show a strong correlation with greater numbers of locations falling into higher probability bins.

### Annual survival

We modeled annual survival rates between melanistic and gray *Canis* taxa using the Kaplan-Meier estimator. To provide robust hazard ratio estimates among melanistic and gray *Canis*, we used a Cox proportional hazard model [93] with coat color as a dummy variable (melanistic = 1, gray = 0). We used a right-censored design with time-at-risk based on time (days) since the animal’s first capture [94] and evaluated main effects only. We tested the proportional hazards assumption of Cox PH using the formal test recommended by [93] and found no significant violations of proportionality in any of the predictor variables included in models (all *P* > 0.05). We used a 365-day (recurrent) time scale to model the baseline hazard [94], standardized to a year beginning on 1 January and ending on 31 December.

## Data Availability

The datasets generated during the current study are available from the corresponding author on reasonable request.

## Abbreviations

USFWS: United States Fish and Wildlife Service
GPS: Global Positioning System
PCA: Principal component analysis
PC: Principal component
GLMM: Generalized linear mixed model
SD: Standard deviation
AIC: Akaike information criterion
SRA: Savannah River area
VHF: Very-high frequency
REML: Restricted maximum-likelihood
dBBMM: Dynamic Brownian bridge movement model
RSF: Resource selection function
USGS: United States Geological Survey
NLCD: National Land Cover Database
